# Prevalence of rifampicin resistance tuberculosis among HIV/TB coinfected patients in Benue State, Nigeria

**DOI:** 10.11604/pamj.2021.38.203.19034

**Published:** 2021-02-23

**Authors:** Francis Enenche Ejeh, Ann Undiandeye, Kenneth Okon, Kazeem Haruna Moshood

**Affiliations:** 1 Department of Veterinary Microbiology, University of Maiduguri, Maiduguri, Nigeria,; 2 Federal Medical Center, Yola, Adamawa State, Nigeria,; 3 Department of Medical Microbiology, Federal Medical Centre, Makurdi, Nigeria,; 4 Department of Veterinary Microbiology, Ahmadu Bello University, Zaria, Nigeria

**Keywords:** Microbiology, drug resistance, infection control

## Abstract

**Introduction:**

the emergence of HIV/TB co-infection has changed the global health landscape globally, particularly in sub-Saharan Africa and Asia with a high prevalence rate. It has further worsened and compound patient diagnosis, treatment/management approach and infection control. Rifampicin resistance TB (RR-TB) is a good indicator of treatment failure and infection control in the community. This study determines the prevalence of RR-TB among HIV/TB coinfected patients in Benue State, Nigeria.

**Methods:**

the case-control study was carried out at Federal Medical Centre, Makurdi and General Hospital, Otupko, between January 2017 and February 2018. One thousand and ten suspected tuberculosis and HIV patients were enrolled in the study, diagnosed according to WHO guidelines. Sputum samples were collected and then analyzed by acid-fast bacilli smear test and GeneXpert MTB/RIF assay.

**Results:**

overall prevalence of tuberculosis by acid-fast test was 74 (7.3%), 171 (16.93%) by GeneXpert assay and 2.18% by RR-TB test respectively. Significant difference was observed between the detection technique and demographic variables, high prevalence among urban patient compared to rural (8.85%vs 5.40%; X^2^= 4.38; P = 0.036) and ethnic background of the patients (X^2^= 23.21; P = 0.000) by acid fast test. With GeneXpert, high prevalence recorded among patient within age-group15-45years (X^2^= 8.01; P = 0.046) and ethnic group (X^2^= 6.30; P = 0.044). The occurrence of HIV/TB co-infection was less associated with Idoma ethnic group (COR; 0.440; 95% C.I; 0.246 - 0.786).

**Conclusion:**

the relatively high prevalence of HIV/TB co-infection and RR-TB is a tremendous public health threat, considering society's attendant implication. Further surveillance studies are needed to evaluate the situation in Benue State better.

## Introduction

*Mycobacterium tuberculosis (M. tuberculosis)* is a member of the group *Mycobacterium tuberculosis* complex (MTBC), the causative agent of tuberculosis (TB) in humans and animals [1-3]. TB and HIV comorbidity of high mortality rate in sub-Saharan Africa. The emergence of drug-resistant TB, such as multidrug-resistant (MDR) and extensively drug-resistant (XDR) TB, is the hindering factors militating against the effective control of TB worldwide [4]. The World Health Organization recommends continuous surveillance and documentation of TB and rifampicin resistance tuberculosis (RR-TB) prevalence among high-risk populations. TB is ranked above HIV/AIDS as the leading cause of death, rendering it an alarming public health issue globally. Globally, about 9 million new cases and 1.5 million deaths have resulted from TB annually [4]. The majority of new TB cases and TB elicited fatalities occur in developing countries, such as Nigeria [5, 6]. The emergence of HIV/AIDS pandemic is a major contributing factor in the global increase of TB incidence [7], particularly among more impoverished populations. Nigeria has an estimated national adult HIV prevalence of 3.6% [8], with 3.3 million people living with HIV and represent the second-highest burden of HIV in Africa. Benue State is at high risk for TB burden in Nigeria due to its high prevalence of HIV/AIDS (12.7%) [8]. Additionally, it has been reported that there is a strong relationship between drug resistance TB and HIV/AIDS in Nigeria [9, 10].

Nigeria ranked fourth among twenty-two high TB burden countries with 586,000 (345,000 -890,000) incidence cases and 100,000 (56,000 -155,000) HIV positive incident TB cases [6]. Nigeria not only has a surprisingly high death rate (44/100,00) of TB affecting HIV positive individuals, but it is also the country with the highest death rate (97/100,000) for individuals negative for HIV [6]. TB has become an epidemic that has extended itself into every part of Nigeria. In Kano, northwest Nigeria, 14.7% prevalence was reported among patients attending infectious disease hospital [11]. HIV/TB co-infection prevalence of 17.2% in Southeast Nigeria [12] and 24.8% in Calabar, the Niger Delta region [13]. Benue State has a high TB burden attributed to a high HIV/AIDS [14]. Anígilájé *et al*. [15] reported a 19.8% prevalence of TB among HIV infected children in Benue State, while Nwadioha *et al*. [16] reported 21.5% prevalence of TB among patients in Makurdi Benue State.

The gold standard for diagnosing TB is the detection of *Mycobacterium tuberculosis* by culture and biochemical identification. However, this method is time-consuming, requiring special laboratory and expertise [4]. GeneXpert MTB/RIF assay´s development and its endorsement by the World Health Organization (WHO) to implement national TB programs in developing countries has dramatically reduced TB diagnostic period and improved TB treatment and management [17-19]. The GeneXpert is an automated technology that rapidly and simultaneously processes sputum specimen and detects MTBC and rifampicin resistance mutation in the rpoB gene. This assay was approved and recommended for TB diagnosis by WHO especially suspected HIV/TB comorbidity globally [20, 21]. There is the paucity of information on the prevalence of TB and RR-TB in Benue State. Rifampicin resistance-TB provides adequate information on patients' adherence to TB regimen and template for effective infection control. Therefore, the study determines the prevalence of tuberculosis and RR-TB among HIV positive patients in Benue State, Nigeria.

## Methods

**Study setting:** this descriptive case-control study was conducted at the directly observed therapy (DOTS) at the Federal Medical Centre (FMC), Makurdi and the General hospital, Otupko. The study spanned between January 2017 and February 2018. The study participants comprised 332 HIV seropositive and 678 HIV seronegative patients. The study was approved by the Research and Ethics Committee of the Federal Medical Center, Makurdi and the Health Research Ethics Committee, Benue State University Teaching Hospital, Makurdi, Nigeria. The study questionnaire and consent form were administered to patients willing to participate in the study after a verbal briefing on the study's public health importance. The questionnaire's information includes age, gender, ethnic background, residential location, TB, and HIV status.

**Sputum sample collection:** each patient was asked to provide two sputum samples after giving consent to participate in the study. The first sputum sample was collected upon enrollment, and the second was collected the following morning. A physician did not induce sputum collections. The first sputum sample was tested with GeneXpert *Mycobacterium tuberculosis*/resistance to rifampicin (MTB/RIF), while the second sample was subjected to AFB smear microscopy. These procedures were carried out at the TB unit of the Microbiology Department at the FMC, Makurdi and the General Hospital, Otukpo.

**Microscopic examination of sputum samples:** conventional Ziehl-Neelsen (ZN) was performed to investigate the presence of acid-fast bacilli (AFB). Slides showing pink/red coloured acid-fast bacilli were recorded as positive. The procedure was done according to a previous report [22].

**GeneXpert MTB/RIF PCR test:** GeneXpert MTB/RIF polymerase chain reaction (PCR) tests were carried out according to manufacturer´s instructions. Sample reagent was added to untreated sputum at a 2:1 ratio. The mixture was manually agitated twice during a 15minute incubation period at room temperature. 2 ml of the mixture were transferred into a test cartridge with a sterile pipette. The cartridge was then loaded into the GeneXpert machine. GeneXpert MTB/RIF PCR test results were received through computer software and subsequently printed for filing at the end of the real-time PCR assay.

**Statistical analysis:** data obtained were analyzed using the Chi-square test; logistic regression analysis was used to clarify the predictors for HIV/TB co-infection and rifampicin resistance. P values were two-tailed, with P < 0.05 being considered statistically significant. Statistical analyses were performed using SPSS 20.0 (SPSS Inc. Chicago, IL, USA).

**Ethical approval:** the permission (BSUTH/MKD/HREC2013B/2017/0011) to conduct this study was obtained from the Health Research Ethics Committee, Benue State University Teaching Hospital, Makurdi, Nigeria. There is no way to trace patients' identities from the results generated in this study.

## Results

The overall prevalence of tuberculosis by Smear microscopy (AFB) was 7.33% (73/1010). The prevalence among age groups was not significantly different (X^2^ = 4.316; P = 0.229). However, patients within the age of 15 -34 had the highest prevalence (9.33%) of TB than other age groups. Patients from urban areas had significantly (X^2^ = 4.38; P = 0.036) higher prevalence of TB than patients from rural areas (8.85% vs 5.40%). There was significant (X^2^= 23.21; P = 0.000) difference between ethnic groups in the study areas. Tiv (11.08%) and other (15.25%) ethnic groups had a higher prevalence of TB than patients from the Idoma ethnic group (3.91%). Patients who were diagnosed at FMC, Makurdi had significantly (X^2^ = 22.30; P = 0.000) higher prevalence of TB than patients who visited General hospital, Otukpo (12.04% vs 4.15%) (Table 1).

**Table 1:** comparative detection of tuberculosis by smear microscopy and GeneXpert in Benue State

		Smear Microscopy n = 1010				GeneXpert n = 1010			
Variables	No Sampled	No Positive	(%)	χ^2^	P-Value	No Positive	(%)	χ^2^	P- Value
**Gender**									
Male	498	40	8.03	0.72	0.396	92	18.47	1.66	0.209
Female	512	34	6.64			79	15.43		
**HIV Status**									
HIV +	331	36	7.85	0.202	0.653	53	16.01	0.30	0.655
HIV −	679	48	7.07			118	17.38		
**Age group**									
0-14	64	4	6.25	4.316	0.229	8	10.25	**8.01**	**0.046**
15-34	418	39	9.33			77	18.42		
35-54	375	23	6.13			71	18.93		
≥55	152	8	5.26			15	9.87		
**Address**									
Urban	565	50	8.85	**4.38**	**0.036**	100	17.70	0.54	0.464
Rural	445	24	5.40			71	15.96		
**Ethnicity**									
Tiv	388	43	11.08	**23.21**	**0.000**	63	16.24	**6.30**	**0.044**
Idoma	563	22	3.91			91	16.16		
Others	59	9	15.25			17	28.81		
**Hospital**									
FMC MKD	407	49	12.04	**22.30**	**0.000**	71	17.44	0.13	0.720
GH OTKP	603	25	4.15			100	16.58		
									
**Total**	**1010**	**74**	**7.33**			**171**	**16.93**		

FMC MKD: Federal Medical Centre, Makurdi GH OTKP: General Hospital, Otukpo

Overall prevalence of TB in the study population by GeneXpert assay was 16.93% (171/1010). Of these, patients within the age bracket 15 -34 (18.42%) and 35 -54 had higher prevalence of TB than those within 0 -14 (10.25%) and 55 years and above (9.87%). The difference in TB prevalence among the age groups was significant (X^2^ = 8.01%; P = 0.046). Patients from urban areas had a higher TB prevalence (17.70%) by GeneXpert assay than patients from rural areas. However, the difference was not statistically significant (X^2^ = 0.54; P = 0.464). The Idoma and Tiv (16.24% and 16.16%) had about the same TB prevalence; other ethnic groups had the highest prevalence (28.81%). Also, the difference in TB prevalence among the different ethnic groups in the study population was statistically significant (X^2^ = 6.30; P = 0.044) (Table 1).

Both crude and adjusted odds ratio were higher for HIV/TB coinfected patients within the age bracket 35 -54 (COR 1.963, 95% CI 0.796 -4.842; AOR 2.154, 95% CI 0.865 -5.366) than other age groups. The Idoma ethnic group had significantly (P = 0.006) lower crude odds ratio (COR 0.440, 95% CI 0.246 -0.786) for HIV/TB co-infection than Tiv and other ethnic groups. Patients diagnosed at FMC, Makurdi had a higher crude and adjusted odds ratio for HIV/TB co-infection than patients who visited General hospital, Otukpo (Table 2).

**Table 2:** HIV/TB co-infection among patients who attended DOTS Centers in Makurdi and Otukpo, Benue State

Variable	HIV/TB co-infection (%)	COR (95% CI) P -Value	AOR (95% CI) P -Value
**Sex**			
Male	26 (48.1)	1.00 (ref.)	1.00 (ref.)
Female	28 (51.9)	1.050 (0.607-1.818) 0.861	0.983 (0.562-1.719) 0.951
**Age Group**			
0-14	3 (5.6)	1.197 (029-4.940) 0.804	1.470 (0.347-6.216) 0.601
15-34	17 (31.5)	1.032 (0.399-2.667) 0.949	1.276 (0.487-3.347) 0.620
35-54	28 (51.9)	1.963 (0.796-4.842) 0.143	2.154(0.865-5.366) 0.099
≥ 55	6 (11.1)	1.00 (ref.)	1.00 (ref.)
**Address**			
Urban	35 (64.8)	1.481 (0.835-2.626) 0.179	0.849(0.447-1.610) 0.615
Rural	19 (35.2)	1.00 (ref.)	1 (ref.)
**Ethnic Group**			
Tiv	30 (55.6)	1.00 (ref.)	1.00 (ref.)
Idoma	20 (37.0)	**0.440 (0.246-0.786) 0.006**	0.544 (0.173-1.710) 0.297
Others	4 (7.4)	0.868 (0.294-2.559) 0.797	0.789 (0.242-2.566) 0.693
**Hospital**			
FMC MKD	**37 (68.5)**	**3.447 (1.913-6.211) 0.000**	**4.767 (1.941-11.703) 0.001**
GH OTKP	17 (31.5)	1.00 (ref.)	1.00 (ref.)
**Total**	**54 (100)**		

The overall prevalence of rifampicin resistance tuberculosis in the study population was 2.18%. However, not statistically significant, crude and adjusted odds ratio indicated that HIV patients were less likely to have rifampicin resistance tuberculosis than non-HIV patients (COR 0.578, 95% CI 0.247 -1.351; AOR 0.642, 95% CI 0.245 -1.681). Patients within the age group 15 -34 and 35 -54 were twice as likely to have rifampicin resistance tuberculosis (Table 3). Also, the adjusted odds ratio indicated that patients diagnosed at FMC, Makurdi, were twice as likely to have rifampicin resistance tuberculosis than those diagnosed at the General hospital, Otukpo (Table 3).

**Table 3:** rate of detection of rifampicin resistance among different populations in Benue State

Variable	Total	Positive (%)		P - value	COR (95% CI) P- Value	AOR (95% CI) P-Value
**Gender**						
Male	498	12 (2.41)	0.247	0.671	1.00 (ref.)	1.00 (ref.)
Female	512	10 (1.95)			1.240 (0.531-2.895) 0.620	1.325 (0.561-3.128) 0.521
**HIV Status**						
HIV +	331	10 (3.02)	1.642	0.200	0.578 (0.247-1.351) 0.205	0.642 (0.245-1.681) 0.367
HIV −	679	12 (1.77)			1.00 (ref.)	1.00 (ref.)
**Age group**						
0-14	64	0 (0.00)	2.443	0.486	1.00 (ref.)	1.00 (ref.)
15-34	418	11 (2.63)			2.027 (0.444-9.252) 0.362	2.142 (0.459-9.991) 0.332
35-54	375	9 (2.40)			1.884 (0.394-8.636) 0.437	1.832 (0.384-8.737) 0.447
≥55	152	2 (1.32)			0.000 (0.00-0.0) 0.997	0.000 (0.00-0.0) 0.997
**Address**						
Urban	565	12 (2.12)	0.018	0.894	0.9 (0.404 -2.205) 0.894	0.748 (0.296 -1.888) 0.539
Rural	445	10 (2.25)			1.00 (ref.)	1.00 (ref.)
**Ethnicity**						
Tiv	388	8 (2.06)	0.437	0.862	0.600 (0.124-2.896) 0.525	0.336 (0.064-1.771) 0.198
Idoma	563	12 (2.13)			0.621 (0.136-2.842) 0.539	0.824 (0.163-4.169) 0.815
Others	59	2 (3.39)			1.00 (ref.)	1.00 (ref.)
**Hospital**						
FMC MKD	407	11 (2.70)	0.880	0.348	1.495 (0.642-3.481) 0.351	2. 763 (0.721-10.589) 0.138
GH OTKP	603	11 (1.82)			1.00 (ref.)	1.00 (ref.)
**TOTAL**	**1010**	**22 (2.18)**				

## Discussion

The overall prevalence of TB of 7.33% by smear microscopy and 16.93% by GeneXpert MTB/RIF affirmed the findings that GeneXpert has high sensitivity and specificity in detecting TB infection in the clinical specimen as documented in other studies [20, 23-25]. The prevalence reported in this study was consistent with previous studies [17, 18, 26]. The high sensitivity and specificity of GeneXpert assay in detection of TB infection collaborated with a study among HIV/TB co-infection and RR-TB conducted in South Africa with a prevalence of 17.3% Lawn *et al*. [27]. Similarly, Habte *et al*. [28] report GeneXpert MTB/RIF detection rate of (35.9%) cases of TB compared to 12.8% by smear microscopy and stated that GeneXpert helped in household contact tracing [28].

There was no significant difference in TB prevalence between HIV positive and HIV negative patients in this present study. In contrast, Babatunde *et al*. [12] reported a higher prevalence of TB among HIV negative patients than in HIV positive patients. A study conducted in Ethiopia reported a significantly higher prevalence of TB among HIV positive than HIV negative patients [29]. A significant difference was observed with the age group, comparing the patients' detection methods and demographic variables and demographic variables. There was a high HIV/TB prevalence within 15 -34 years and 35 to 54 years. This result was consistent with the findings of previous reports in Nigeria [14, 30]. The high prevalence of TB and HIV/TB co-infection among these age groups may be due to the high cases of HIV [8, 31-33]. Additionally, Ojiezeh *et al*. [14] reported a high prevalence of HIV/TB co-infection among sexually active, productive/childbearing persons aged 34-44. Primarily due to social-cultural practices that were predisposing them to this infection. The patients' residential location varies with TB prevalence and detection methods. The prevalence of TB was significantly high among patients in urban areas than those in rural areas, attributable to the variances in population density and access to healthcare facilities for proper diagnosis [34]. Prasad *et al*. [35] stated that two factors are responsible for a higher prevalence of TB in urban areas than in rural areas. Firstly, a high proportion of urban residents in low and middle-income countries live in dismal conditions that consist of overcrowding, poor-quality housing, and lack of water sanitation and secure tenure. Secondly, there is abundant inequality in health care access and quality in some urban areas.

We observed that the prevalence of TB was significantly different among ethnic groups in Benue State. The Idoma ethnic group had a lower TB prevalence than Tiv and other ethnic groups, as indicated by both smear microscopy and GeneXpert. Also, the Idoma ethnic group had a significantly lower risk of acquiring HIV/TB co-infection. The differences in the prevalence and risk of TB and HIV/TB co-infection among different ethnic groups in Benue State could be attributed to differences in socio-cultural practice that predispose them to infection. This observation coincided with a similar study conducted in Ethiopia in which the ethnic Afar population had a relatively low TB prevalence than other ethnic groups [29]. The relatively high prevalence of TB among the Tiv and other ethnic groups was worrisome for reasons stemming from TB transmission and poor ventilation, and overcrowding. The Tiv ethnic group´s observed prevalence might be primarily due to their occupational activities as they are an agrarian community known for their large family sizes. Every member contributes to the upkeep and arduous labour of maintaining the family farm. The communal lifestyle, residing within a densely populated household with poor ventilation, facilitates TB transmission [36, 37]. There was high HIV among the Tiv ethnic group [38], hence the high HIV associated TB [39]. Among the other ethnic groups, the Hausa are the majority. The Hausa ethnic group lives in densely populated urban poor areas in Benue State. Their practice of polygamy characterizes large family sizes that lead to overcrowding and poor nutrition [40].

Patients who attended the FMC of Makurdi had a higher prevalence of TB than those who attended the General Hospital of Otukpo. We also observed that patient who visited the FMC of Makurdi had a higher risk of HIV/TB co-infection. The high prevalence of TB and high risk of HIV/TB co-infection among patients who attended the FMC, Makurdi can be explained by the FMC's role as a referral centre. Therefore, it receives more positive cases of TB and HIV/TB co-infection than the General Hospital, Otukpo. Furthermore, literacy levels and work experience are essential in the performance of TB diagnostic testing. The FMC has a higher pay rate and a greater number of laboratory staff than the Benue State Ministry of Health. There are more qualified and educated personnel at the FMC of Makurdi than at the Otukpo General Hospital.

In this study, we reported a prevalence of 2.2% rifampicin resistance among HIV and non-HIV positive TB patients who attended TB clinics in both the FMC of Makurdi and the General Hospital of Otukpo. The results obtained in this study were lower than previous studies in Southwest Nigeria (5.5%) [41]. In another research study conducted in three Nigerian cities, 23% prevalence and 11% rifampicin resistance were reported among failed and new treatment cases. A higher prevalence of rifampicin resistance was also reported in Ethiopia [42, 43]. The prevalence of rifampicin resistance reported in this study was consistent with WHO's estimated prevalence of 2.2% [7]. The low prevalence of rifampicin resistance observed in this study compared with other research in Nigeria and Ethiopia may be due to differences in sample size, location and other factors that were not considered in this study. Moreover, Mulu *et al*. [43] explained that variation in the rate of rifampicin resistance among different authors could be due to differences in the risk for HIV acquisition, exposure to anti-TB drugs and national TB control programs.

In this study, we observed that gender, HIV status, age and residence in urban versus rural areas were not significant factors that could influence rifampicin resistance. This finding agreed with the report of Masenga *et al*. [44]. Masenga *et al*. [44] explained that both males and females had equal exposure to rifampicin resistance causative factors. A similar trend was reported among previously treated and new cases of TB in Ethiopia [43].

## Conclusion

The relatively high prevalence of HIV/TB co-infection is of public health concern, considering the consequential impact on the community, as high prevalence was observed among the sexually active age groups. The RR-TB prevalence of 2.2% is an indicator of the emerging TB patient treatment failure trend, which could further worsen the infection control strategy. Future studies are needed for better understanding of the TB epidemiology and infection control intervention.

### What is known about this topic

- The prevalence of tuberculosis in Benue State is high because of the high burden of HIV/AIDS;
- There is poor tuberculosis treatment outcome due to HIV/AIDS and TB comorbidity;
- Tuberculosis treatment and diagnostic centres are located in each senatorial district in Benue state, but there is the paucity of published data on the prevalence of tuberculosis and rifampicin-resistant tuberculosis.

### What this study adds

- The overall prevalence of tuberculosis in the study area was 7.33% and 16.93% by acid-fast microscopy and GenXpert MTB/RIF;
- The prevalence of TB was higher among patients from urban areas than from rural dwellers (8.85% vs 4.28%);
- The prevalence of rifampicin resistant tuberculosis among the study population was 2.18%.
